# Reliability of the Polish version of the Overactive Bladder Symptom Score (OABSS) questionnaire: Correlation of the OABSS with urodynamic study and the UDI-6 and IIQ-7 questionnaires

**DOI:** 10.1007/s00192-019-04060-2

**Published:** 2019-08-08

**Authors:** Andrzej Wróbel, Katarzyna Skorupska, Ewa Rechberger, Andrzej Woźniak, Pawel Miotla, Agnieszka Kubik-Komar, Pawel Skorupski, Tomasz Rechberger

**Affiliations:** 1 2nd Department of Gynecology, Medical University in Lublin, ul. Jaczewskiego 8, 20-954 Lublin, Poland; 2 Department of Applied Mathematics and Computer Science, University of Life Science, Lublin, Poland

**Keywords:** Mixed urinary incontinence, Overactive bladder, Overactive Bladder Symptom Score, Validation

## Abstract

**Introduction and hypothesis:**

Overactive bladder (OAB) and mixed urinary incontinence (MUI) are significant problems worldwide. Their broad definition makes them difficult to diagnose; therefore, specialists need a tool to confirm diagnosis. The Overactive Bladder Symptom Score (OABSS) is used in the objective diagnosis of OAB. We aimed to develop and evaluate the effectiveness of OABSS for patients in Poland suffering from OAB and MUI and to correlate it with UDI-6 and IIQ-7.

**Methods:**

A total of 824 women suffering from urinary incontinence (UI) aged between 18 and 75 years were included. SUI (*n* = 290); OAB (*n* = 285) and MUI (*n* = 249) were confirmed by medical history and urodynamic study. Of the subjects, 821 women completed the Polish version of OABSS on two separate visits: weeks 0 and 2. In addition, they undertook UDI-6 and IIQ-7 during Week 2. The Cronbach’s alpha (α) was used to estimate the internal consistency. Scores were compared using the intraclass correlation coefficient (ICC).

**Results:**

We observed statistically significant differences (*p* < 0.0005) between mean scores of OABSS among patients from the study groups OAB-SUI and MUI-SUI. We did not observe statistically significant differences between patients from the MUI and OAB groups (*p* > 0.11). Analysis also did not show statistically significant differences between visits.

The internal consistency was very good: α = 0.89 (SUI); = 0.9 (OAB); = 0.82 (MUI). In all groups, test–retest reliability was excellent; ICC was >0.99.

**Conclusions:**

The Polish version of the OABSS is a reliable tool for females suffering from UI. However, OABSS does not distinguish patients with MUI from patients with OAB.

## Introduction

Overactive bladder (OAB) and mixed urinary incontinence (MUI) are significant problems worldwide. Indeed, the prevalence of OAB was expected to increase about 20.1% from 2008 to 2018 [1]. This condition can impair quality of life related to health in terms of decreased work productivity, loss of sleep, depression, diminished sexual health and emotional well-being in women and men [2]. The estimated prevalence of MUI is approximately 30% in all women suffering from incontinence. The broad definition of the term, however, makes it difficult to diagnose [3]. Hence, specialists and non-specialists need a tool to confirm diagnosis and establish treatment that increases urgency control and improves patient quality of life while reducing side effects.

Currently, the diagnostic process of OAB consists of: medical history (MH), physical and neurological examination, questionnaires and/or bladder diary (BD), urine analysis and urodynamic study (UDS) [4]. UDS is an invasive assessment of the lower urinary tract (LUT) that requires trained specialists. Indications for UDS in patients suffering from OAB are, however, disputed. Some researchers state that UDS is necessary for the diagnostic process, as UDS is the only objective assessment of LUT function/dysfunction in incontinent patients; others claim that UDS should be reserved for those with suspected voiding difficulty or neuropathy or who are unresponsive to initial therapy [5]. Since OAB is characterized by individual symptoms (frequency, urgency), rather than objective measurements, a valid way of measuring the patient’s symptoms is essential in the treatment process.

The Overactive Bladder Symptom Score (OABSS) has been developed to express all of the OAB symptoms in a single result. Four symptoms addressing day- and night-time frequency, urgency, and urgency incontinence are scored in the Homma OABSS [6]. The Blaivas OABSS consists of seven questions on a five-point Likert scale that refer to every symptom of OAB: 1 for nocturia, 1 for urinary frequency, 3 for urgency, 1 for urge incontinence, and 1 generic question about bladder control. The total result is scored from 0 to 28, with patients with higher scores reporting worse symptoms. In addition to the total OABSS result, the questionnaire contains an urgency subscale (containing questions from 3 to 6) that grades severity of urgency. Of note, urgency is the basic symptom of OAB, but some authors believe that urgency is an all-or-nothing experience and should not be graded [7]. Other studies have a different opinion and their results show that urgency is subjective, but can be assessed [8, 9].

In our work, the survey was designed to validate the efficacy of the seven-question OABSS for the Polish population by establishing the validity, test–retest reliability and internal consistency of a professionally translated questionnaire. This was done to secure a valid instrument for urinary incontinence (UI) diagnosis and to correlate it with UDS, the Urogenital Distress Inventory (UDI-6), and the Incontinence Impact Questionnaire (IIQ-7).

## Materials and methods

### Translation

Three independent translators translated the original OABSS into Polish. A native speaker back-translated it into English to ensure conceptual equivalence with the original OABSS version. Ten women suffering from UI (confirmed by urodynamics) were tested using the Polish version of the questionnaire. No major issues were observed, and minor problems that arose were straightened out.

### Study population and study design

A total of 824 women with UI aged between 18 and 75 years, who met the inclusion criteria, were chosen from patients attending the Outpatient Clinic between June 2017 and December 2018. After signing the informed consent, socio-demographic data such as age, parity, BMI, and menopausal status were taken. Polish was the native language of all patients within the study population. Of the study group, recruitment medical examination/case history and urodynamic investigation performed during visit week 0 showed that 290 patients had SUI, 285 had OAB, and 249 had MUI. All patients completed the OABSS questionnaire at baseline (visit week 0). Moreover, 821 women repeated the OABSS and 824 completed the UDI-6 and IIQ-7 questionnaires after 14 days. Twenty-three patients who did not reappear in week 2 were excluded from evaluation. None of the patients received treatment between visits. In this part of the study, only the answers of patients who fully completed the questionnaires were taken into account. The local bioethics committee approved the study.

### Sample size

The sample size was established based on Comfrey and Lee’s suggestion that 50 is very poor, 100 is poor, 200 is fair, 300 is good, 500 is very good, and 1,000 or more makes for an excellent sample size [10].

### Validity

Face/content validity was assessed by obtaining feedback from a group of 10 women suffering from UI, and from 3 doctors taking part in the study who were specialists in the urogynecology field. They reviewed the seven-question OABSS questionnaire and clarified the translation.

To evaluate the factor structure and construct the validity of the OABSS, principal component analysis (PCA) using VARIMAX rotation was conducted for all seven questionnaire items. The data from the entire sample group at day 0 were analyzed. PCA results were also confirmed by Spearman’s correlations among OABSS items.

The final Polish version of the OABSS was subsequently administered to Polish-speaking women assessed to have UI at university-based urogynecology clinics in Poland. Polish versions of the UDI-6 and IIQ-7 were also administered to evaluate the criterion validity of the Polish OABSS. The results of UDI-6 and IIQ-7 application were compared using Pearson’s correlation coefficient to assess the criterion validity.

The internal consistency of the OABSS questionnaire was estimated by way of Cronbach’s alpha coefficient (α). Here, a value greater than 0.7 indicates high reliability.

Reliability was tested by applying the intraclass correlation coefficient (ICC), an index or repeatability, by utilizing statistical software R. Here, ICC at ≥0.7 was considered acceptable [11].

### Statistical analysis

Statistical analysis was performed using STATISTICA version 13.1 software (StatSoft, Kraków, Poland) in addition to open source R software [12]. *p* values less than 0.05 were considered significant. Significance of differences of means between studied groups was assessed using one-way ANOVA and the Tukey post hoc test.

## Results

Detailed characterization of patients included in the study is presented in Table 1.

**Table 1:** Demographic characteristics of the patients from the study groups (continuous variables are presented as the mean ± SD, categorical variables are presented as numbers and percentages)

Parameter	Study group (*N* = 821)		
	SUI (*n* = 290)	OAB (*n* = 283)	MUI (*n* = 248)
Age (years), mean ± SD	54.4 ± 10.8	55.5 ± 12.6	53.6 ± 12.4
BMI (kg/m^2^), mean ± SD	26.9 ± 3.9	26.7 ± 5.9	26.5 ± 5.5
Parity, mean ± SD	2.3 ± 0.9	1.8 ± 1.1	2.0 ± 1.1
Postmenopausal, *n* (%)	181 (62.4)	194 (68.5)	163 (65.7)
Parous, *n* (%)	279 (96.2)	273 (96.4)	227 (91.5)

*SUI* stress urinary incontinence, *MUI* mixed urinary incontinence, *OAB* overactive bladder, *BMI* body mass index

The validation process shows that the Polish OABSS version has good face validity—no major troubles arose during the application and most respondents found it “comprehensive” and a “good” measure.

The result of PCA using VARIMAX rotation indicated that the high degree of system variation (almost 80%) is explained by the first two components (F1 and F2). F1 correlates strongly questions 1–6, whereas F2 correlates strongly only with question 7. The reason why not all of the questions constitute the first principal component (F1) was the fact that the last question, unlike the others, concerned the subjective feelings of the patient (Table 2). The results of PCA were also confirmed by Spearman’s correlation coefficient between particular questions. This indicated good construct validity (Table 3).

**Table 2:** Results of principal componence analysis (PCA)—factor loadings after VARIMAX rotation

	F1	F2	F3	F4	F5	F6	F7
OABSS Ques-1-T1	**0.850**	−0.090	−0.342	−0.026	0.368	0.124	0.010
OABSS Ques-2-T1	**0.834**	0.000	−0.275	−0.388	−0.259	−0.103	0.010
OABSS Ques-3-T1	**0.914**	−0.155	0.054	0.178	−0.124	0.083	−0.290
OABSS Ques-4-T1	**0.906**	−0.059	0.062	0.210	−0.199	0.201	0.218
OABSS Ques-5-T1	**0.889**	−0.164	0.065	0.235	0.083	−0.336	0.062
OABSS Ques-6-T1	**0.809**	0.070	0.463	−0.320	0.148	0.039	0.007
OABSS Ques-7-T1	0.397	**0.910**	−0.044	0.108	0.006	−0.024	−0.024
Eigenvalue	4.679	0.895	0.419	0.396	0.286	0.188	0.136
Explained variance (%)	66.841	12.786	5.992	5.658	4.092	2.687	1.944

F1–F7—extracted principal components. Factor loadings values >0.8 are marked

The contribution of individual variables on the construction of a new factor is assessed on the basis of the values of these factors. A Limit value is 0.8 and values above this limit are highlighted

**Table 3:** Spearman’s rank correlation coefficients between OABSS items

	OABSS Ques-1-T1	OABSS Ques-2-T1	OABSS Ques-3-T1	OABSS Ques-4-T1	OABSS Ques-5-T1	OABSS Ques-6-T1
OABSS Ques-2-T1	0.732					
OABSS Ques-3-T1	0.734	0.730				
OABSS Ques-4-T1	0.705	0.717	0.855			
OABSS Ques-5-T1	0.723	0.679	0.817	0.784		
OABSS Ques-6-T1	0.612	0.624	0.693	0.684	0.692	
OABSS Ques-7-T1	0.253	0.276	0.238	0.304	0.215	0.315

In the study, a Pearson’s correlation coefficient between OABSS and UDI-6 of 0.676 and between OABSS and IIQ-7 of 0.545 demonstrates good convergent validity, with high correlation among OABSS items 1 to 6 (*p* < 0.05). Here, item 7 was found to be poorly correlated with the other questions.

The total score of the OABSS ranges from 0 to 28. Table 4 shows mean scores ± SD of the patients’ responses to the questionnaire. We observed statistically significant differences (*p* < 0.0005) between mean scores among patients from the OAB-SUI and the MUI-SUI study groups and no statistically significant differences between patients from the MUI and OAB groups (*p* > 0.11). Moreover, analysis did not register important differences between visits.

**Table 4:** Urogenital Distress Inventory-6 (UDI-6), Incontinence Impact Questionnaire-7 (IIQ-7), and Overactive Bladder Symptom Scores (OABSS) questionnaire scores among patients from the study groups

Questionnaire	SUI			OAB			MUI		
	*n*	Mean	SD	*n*	Mean	SD	*n*	Mean	SD
UDI-6	290	36.9	19.9	285	46.2	18.2	249	55.6	17.8
IIQ-7	290	50.4	17.9	285	60.1	17.5	249	66.6	17.6
OABSS	290	7.9	6.3	283	17.9	6.2	248	16.9	5.4

*SUI* stress urinary incontinence, *MUI* mixed urinary incontinence, *OAB* overactive bladder

For UDI-6, a higher question response value indicates an increased level of disability; in IIQ-7, a higher score denotes diminished quality of life and enhanced severity of symptoms (maximum result of the questionnaire is 100) [13]. Results of UDI-6 and IIQ-7 questionnaires are shown in Table 4.

Table 5 shows the study groups’ α values for the OABSS questionnaire. In all groups, test–retest reliability was excellent. Here, ICC was >0.99.

**Table 5:** The results of internal consistency (Cronbach’s alpha) for Overactive Bladder Symptom Scores (OABSS) in SUI, OAB, and MUI groups of patients

Cronbach’s alpha	Total score	SUI	OAB	MUI
OABSS	0.91	0.89	0.9	0.82

*SUI* stress urinary incontinence, *MUI* mixed urinary incontinence, *OAB* overactive bladder

## Discussion

The OABSS is an instrument used to assess patients with the OAB syndrome. It measures episodes of urination, urgency, and urgency incontinence. It is short (consisting of only seven questions) and simple. The original language version is considered reliable and is widely used. However, until this research, OABSS has never been applied in Poland or translated into Polish. Urgency is one of the symptoms of MUI, therefore, we used the new Polish version of OABSS in an attempt to distinguish between OAB and MUI.

The Scientific Advisory Committee (SAC)—a nonprofit public service organization established to serve as a depository and distributor of high quality, standardized, health outcome measurement instruments—states that any instrument that measures health status must have the characteristics of a valid conceptual and measurement model. These include reliability, validity, responsiveness, interpretability, low respondent and administrative burden, being of comparable alternative forms, and holding appropriate cultural and language adaptation [14].

Reliability is the *internal consistency* or *reproducibility* of research measures.

Internal consistency refers to general agreement between different elements in the same test and is a means of estimating whether several items that purport to measure the same general structure, give similar results [9]. To test for internal consistency, Cronbach’s alpha coefficient (α) is evaluated. Here, α ranges from 0 to 1, and values 0–0.6 prove non-satisfactory reliability, whereas those of 0.6 to 0.7 imply satisfactory, and that of 0.7 to 0.95 denote high reliability. A value of 1 attests to complete agreement—redundancy [15]. We confirmed the high reliability of the test in the UI study population (regardless of UI type), as Cronbach’s alpha of the Polish version of the OABSS is 0.91. Furthermore, analysis of the individual groups (OAB, MUI, SUI) also showed very high (>0.7) reliability (Table 5). As expected, we did find statistically significant differences in answers to OABSS among individual patients. The results indicate that in patients whose score tended to increase, the symptom severity is higher. The highest figure was observed in the OAB group (17.9), whereas patients from the MUI group scored 16.9 and SUI group patients tallied 7.9 points. In original publications, the OABSS questionnaire was tested in patients with confirmed OAB [6, 7] and SUI [6]. MUI patients were not assessed. Our study shows similar answers in patients of the OAB and MUI groups (*p* > 0.05); hence, OABSS cannot distinguish between OAB and MUI.

In addition to the OABSS, the patients of our study completed the UDI-6 and IIQ-7 questionnaires. The results showed statistically significant differences among patients from all study groups. It was previously stated that UDI-6 and IIQ-7 questionnaires are not the best option for confirming MUI diagnosis [16]. Thus, we think that adding UDI-6 and IIQ-7 to the OABSS will not help doctors to distinguish between OAB and MUI.

The International Consultation on Incontinence Modular Questionnaire (ICIQ) Short Form is a diagnostic tool that evaluates the urinary tract symptoms and their influence on the quality of life (QoL) in women and men. Previous studies [16] show its good reliability in the MUI group of patients (0.81); thus, this questionnaire would appear to be a better diagnostic tool in distinguishing between patients with OAB and MUI.

Reproducibility (the possibility of the experiment or study being replicated) was tested by applying the ICC according to McGraw and Wong [11]. The test–retest examination was performed 14 days after week 0. The ICC score of >0.99 revealed in SUI, OAB, and MUI patients excellent agreement among the questionnaire’s questions.

Internal validity or the accuracy of conclusions about whether one variable causes another was assessed using UDS (cystometry and uroflowmetry). We rightfully expected that SUI patients would score better in OABSS than did patients suffering from the other UI types (Table 4).

Based on the results of the study, the Polish version of OABSS is useful, but it cannot distinguish between OAB and MUI. The Polish OABSS is reliable, has very good psychometric validity, and can therefore be used by clinicians for preliminary screening for OAB.

## Conclusion

Urinary incontinence is a significant problem worldwide, and tools such as OABSS can help both specialists and nonspecialists in its diagnosis. OABSS, however, cannot distinguish between OAB and MUI.
